# It’s Just a Phase: Exploring the Relationship Between mRNA, Biomolecular Condensates, and Translational Control

**DOI:** 10.3389/fgene.2022.931220

**Published:** 2022-06-27

**Authors:** Dylan M. Parker, Lindsay P. Winkenbach, Erin Osborne Nishimura

**Affiliations:** ^1^ Department of Biochemistry and Molecular Biology, Colorado State University, Fort Collins, CO, United States; ^2^ Department of Biochemistry, University of Colorado, Boulder, CO, United States

**Keywords:** mRNA localization, translation regulation, biomolecular condensates, phase separation, germ granules, stress granules, P-bodies

## Abstract

Cells spatially organize their molecular components to carry out fundamental biological processes and guide proper development. The spatial organization of RNA within the cell can both promote and result from gene expression regulatory control. Recent studies have demonstrated diverse associations between RNA spatial patterning and translation regulatory control. One form of patterning, compartmentalization in biomolecular condensates, has been of particular interest. Generally, transcripts associated with cytoplasmic biomolecular condensates—such as germ granules, stress granules, and P-bodies—are linked with low translational status. However, recent studies have identified new biomolecular condensates with diverse roles associated with active translation. This review outlines RNA compartmentalization in various condensates that occur in association with repressed or active translational states, highlights recent findings in well-studied condensates, and explores novel condensate behaviors.

## 1 Introduction

### 1.1 mRNAs can Concentrate in Biomolecular Condensates

The spatial organization of cells has fascinated scientists since the advent of the microscope. Observations as early as the 1890s documented dyes concentrating within cytoplasmic aggregates of insect germ cells, structures now known as germ granules (Die, 1890; Voronina et al., 2011). Even as those structures remained mysterious, scientists found evidence of diverse patterns of mRNA accumulation in embryogenesis, neurobiology, and yeast mating-type switching (Rebagliati et al., 1985; Lehmann and Nüsslein-Volhard, 1986; Garner et al., 1988; Long et al., 1997). The mRNAs localized in those studies are now classic models of mRNA transport and localization.

As dozens of large, micrometer-scale ribonucleoprotein (RNP) aggregates similar to those described in the germline—were characterized, focus returned to these mysterious germline granules. Seminal studies determined that germ granules and similar RNP structures are often phase-separated organelles, coined biomolecular condensates (Brangwynne et al., 2009; Kroschwald et al., 2015; Lin et al., 2015; Molliex et al., 2015; Nott et al., 2015). Biomolecular condensates are membraneless organelles that phase separate from the surrounding substrate when weak, multivalent interactions of their components create liquid-liquid, liquid-gel, or liquid-crystalline partitioning (Gifford et al., 1982; Wolf et al., 1999; Han et al., 2012; Li et al., 2012; Hubstenberger et al., 2013; Kroschwald et al., 2015; Olafson et al., 2015). This phase separation gives biomolecular condensates unique physical traits. Molecules can typically exchange between the high concentration condensate and the surrounding low concentration environment, promoting dynamic biological processes (Nott et al., 2015). Due to the physical principles underlying phase separation, condensates can also rapidly buffer cellular concentrations of given components with little to no energetic penalty, adjusting their size to maintain a constant concentration of constituent components in the surroundings (Alberti et al., 2019). Condensation can even segregate components based on features such as electrostatic charge distributions, binding partners, molecular valency, and more (Brangwynne et al., 2009; Kroschwald et al., 2015; Lin et al., 2015; Molliex et al., 2015; Nott et al., 2015). Because condensates represent such a unique cellular environment, understanding their composition is of great interest.

Biomolecular condensates typically contain RNA and protein. As RNA detection methods improved, so have observations of mRNA accumulated within condensates. Great efforts have been made to identify the components of condensates through unbiased and candidate-based screens as well as by isolating condensates (Jain et al., 2016; Hubstenberger et al., 2017; Khong et al., 2017; Youn et al., 2018; Lee et al., 2020a). However, the mere presence of proteins and RNA concentrating in large clusters is not sufficient to call them biomolecular condensates, as their phase-separated state must be verified through experimentation. These experiments typically test whether the RNA-protein aggregates behave as liquids or gels based on their periodic fusing or separation, are dissolvable under certain concentrations of solvents, and that their components mix freely with the surrounding environment (Alberti et al., 2019; Ganser and Myong, 2020). Importantly, the interpretation of these experiments takes careful consideration as other types of interactions can appear deceptively similar to phase separation (McSwiggen et al., 2019). For example, some diffraction-limited RNP foci can appear spherical, have similar recovery dynamics after photobleaching, and dissociate in the presence of aliphatic solvents all while undergoing standard monophasic molecular interactions (McSwiggen et al., 2019). Therefore, it is essential to rigorously determine whether RNP foci are biomolecular condensates using multiple carefully designed assays, as described by McSwiggen et al. (2019). Even once condensate behavior is established, determining the functional roles of condensates is challenging.

### 1.2 The Functions of mRNA Condensation Remain Ambiguous

In recent years, many biological fields have found biomolecular condensates in their systems resulting in diverse posited functional roles for these structures. Biomolecular condensates of the nucleus—the nucleolus, Cajal bodies, and nuclear speckles—are associated with coordination of ribosome assembly, RNA processing, enhancing gene expression, or still unresolved functions, respectively (Politz et al., 2006; Yao et al., 2019; Liao and Regev, 2020; Alexander et al., 2021; Courchaine et al., 2021). Those of the cytoplasm—P-bodies, stress granules, germ granules, and Balbiani bodies—are sites of mRNA metabolism, sequestration, regulatory control, or serve to bring mRNAs, proteins, and organelles together, respectively (Kedersha et al., 2005; Brangwynne et al., 2009; Voronina et al., 2011; Kroschwald et al., 2015; Boke et al., 2016; Protter and Parker, 2016). Beyond these well-studied condensates, additional novel condensates with diverse functional roles are being discovered rapidly (Section 3).

Though experimental data can indicate the function of a biomolecular condensate, it is rare to find experimental evidence that definitively demonstrates condensation is essential for a proposed functional role. One reason for the challenge in definitively assigning functional consequences to condensation is the difficulty in separating the effects of their physical disruption from the perturbation of their components. Often, the elements that promote condensate formation are inseparable from elements proposed to perform functions within them. For instance, depletion of the stress granule proteins TIA-1 and TIAR-1 reduce stress granule formation under many conditions, which correlates with decreased cell viability after stress (Gilks et al., 2004; Eisinger-Mathason et al., 2008; Huelgas-Morales et al., 2016). However, it is difficult to delineate to what extent this is due to the loss of stress granules or loss of TIA-1/TIAR-1 functions directly. Furthermore, condensate nucleation is typically promoted by multiple scaffolds making it difficult to confidently ablate the condensate (Gilks et al., 2004; Jain et al., 2016; Rao and Parker, 2017). Even in conditions where it can be reasonably determined that condensates are eliminated, it is possible that submicroscopic assemblies can form and perform the function ascribed to the condensate, as is the case with stress granules and P-bodies (Rao and Parker, 2017; Youn et al., 2018). These complications have made unambiguously determining the function of condensates a problematic but tantalizing goal.

While it is tempting to hypothesize that RNA condensation always occurs for some purpose, in many instances it remains a formal possibility that condensation is a downstream consequence of gene regulatory processes. For example, mutations that perturb germ granule formation in *C. elegans* often lead to sterility; however, circumstances exist where fertility does not depend on germ granule formation (Gallo et al., 2010). This possibility is supported by a growing body of evidence demonstrating that RNA has a natural tendency to aggregate or phase separate, both in the presence and absence of protein (Tauber et al., 2020; Ripin and Parker, 2021). These findings have given rise to the hypothesis that the functions ascribed to condensates are evolutionary adaptations to the physical tendency of RNA to condense.

The exact functions of RNA condensation itself continues to remain ambiguous, though a large and growing number of biomolecular condensates that concentrate RNA have been studied. A picture emerges that some biomolecular condensates are associated with lowly translated RNAs either sequestered due to their low translational status, sequestered for the purpose of repressing their translation, or a mix of both. In contrast, other biomolecular condensates are associated with active translation, sometimes for the purpose of pushing the nascent protein towards a desired fate. This review will explore and contrast these two types of biomolecular condensates.

## 2 Stress Granules, P-Bodies, and Germ Granules: Biomolecular Condensates Linked to Translation Repression

### 2.1 Stress Granules, P-Bodies, and Germ Granules Function in RNA Metabolism

The three major cytoplasmic biomolecular condensates that concentrate RNA and proteins are stress granules, P-bodies, and germ granules.

Stress granules were first observed by microscopy and cell fractionation, being described as membraneless, phase-dense granules of heat shock proteins formed under stress, which dissipated upon recovery (Nover et al., 1983; Collier and Schlesinger, 1986; Collier et al., 1988). Over time, it was discovered that stress granules form under conditions that inhibit translation initiation throughout eukaryotes (Kedersha et al., 1999). Detailed characterizations found stress granule components vary with different stresses but generally contain stalled 48s pre-initiation complex-associated RNAs, translation regulatory RBPs, and various metabolic enzymes (Kedersha et al., 1999; Jain et al., 2016; Khong et al., 2017). One response to stress is a disruption of nucleocytoplasmic transport that occurs as stress granules sequester key transport components. Upon stress induction, mutants deficient in stress granule formation inappropriately continue nucleocytopasmic transport as well as increase in their abundance of stress-granule-associated RNAs. Yang et al. (2014); Zhang et al. (2018a). Ultimately, these behaviors suggest stress granules store and regulate temporarily translationally repressed mRNA (Protter and Parker, 2016; Youn et al., 2019; Riggs et al., 2020), leading to a “triage” model of stress granule function. The triage model suggests that stress induces coalescence of stress granule RNAs and proteins, leading to intra- and intermolecular remodeling, modification, transcription regulation, and in some instances, the passage of RNAs to the cellular decay machinery. Ultimately, this allows stress granules to reprogram cellular gene expression under duress, as evidenced by the decreased recovery of stress granule defective cells (Takahashi et al., 2013; Yang et al., 2014; Huelgas-Morales et al., 2016; Orrù et al., 2016).

In contrast, Processing-bodies, or P-bodies (not to be confused with P granules), were first observed as fluorescent foci of the mouse 5′–3′ exonuclease, XRN-1 (Bashkirov et al., 1997). As further RNA decay factors, such as the decapping factors and Lsm proteins, were identified in foci, the hypothesis arose that P-bodies are discrete RNA decay factories (Ingelfinger et al., 2002; Fenger-Grøn et al., 2005). The simple model of P-bodies as sites of RNA decay was complicated by the discovery that decay may actually be slowed in P-bodies and can even occur normally in mutants where microscopic P-bodies are not formed (Eulalio et al., 2017; Hubstenberger et al., 2017; Wang et al., 2018). Moreover, the presence of miRNA-mediated translational silencing machinery, cell signaling components, and nonsense-mediated decay inhibitors within P-bodies has suggested a broader role in translation regulation and cellular metabolism (Hubstenberger et al., 2017; D’Lima et al., 2017). While their exact roles remain contested, the fact remains that P-bodies are associated with translational repression, mRNA metabolism, and mRNA decay (Toombs et al., 2017; Zhang et al., 2018b; Suen et al., 2020).

Germ granules across the animal kingdom play widespread roles in RNA regulation. As one of the earliest observed membraneless organelles, the role of germ granules has been intently explored (Die, 1890). Seminal studies in *Drosophila* demonstrated that irradiation of the germ granule-containing posterior pole plasm abolishes germline development but can be rescued by injection of healthy pole plasm (Okada et al., 1974). In contrast, injection of germplasm at the anterior is sufficient to ectopically specify posterior structures in place of normal anterior structures (Illmensee and Mahowald, 1974). Further characterization demonstrated that germ granules play a role in defining the germline transcriptome through multiple mechanisms, including repression of the somatic transcriptional program, suppression of mutagenic transposable elements, and maintenance of germline-specific small RNA surveillance (Voronina et al., 2011). Together, these findings led to a model where germ granules promote germline specification in early development through the orchestration of a germline-specific transcriptome. Even so, demonstrating the functional roles of germ granules has remained challenging. Because germ granules are not strictly required for germ cell specification and due to the challenges in unambiguously demonstrating the modes of regulation occurring within germ granules, tractable model systems for the study of these condensates have proven indispensable.

### 2.2 P Granules: A Model Condensate

**FIGURE 1:**
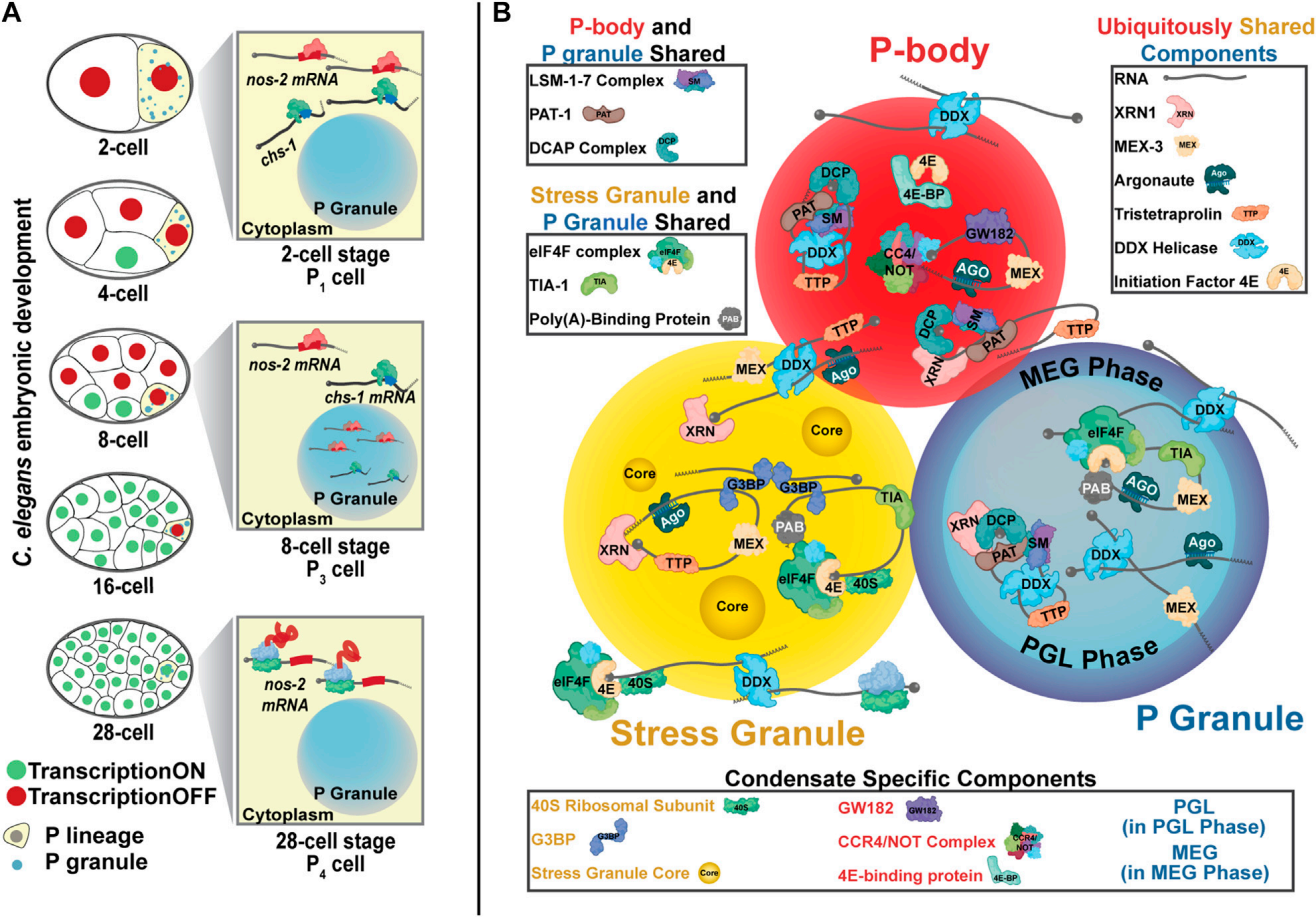
P granules are nematode germ granules. **(A)** *C. elegans* P granules successively concentrate in the posterior P cells that eventually giving rise to the germline. *nos-2* mRNA is found in the cytoplasm of 2-cell stage embryos in a translationally repressed state. From the 4-cell stage to the 28-cell stage, *nos-2* mRNA concentrates into P granules though many *nos-2* mRNA molecules also reside in the cytoplasm. *nos-2* mRNA in P cells is spared from the degradation seen in somatic cells accounting for its concentration down the P lineage. At the 28-cell stage, *nos-2* mRNA emerges from P granules and is translated. *chs-1* mRNA also accumulates in P granules in a manner similar to *nos-2* but is rapidly degraded. **(B)** Three cytoplasmic biomolecular condensates—P granules (germ granules), P-bodies, and stress granules—share key components and have some overlapping functions. Some proteins are distinct to each condensate. The components here represent diverse organisms and are not exhaustive.

The P granules of *Caenorhabditis elegans* were among the first membraneless organelles recognized as phase-separated condensates (Brangwynne et al., 2009). P granules, the nematode germ granules, concentrate through the progenitor germ lineage contributing to gamete production and fertility in adults (Kawasaki et al., 2004; Batista et al., 2008; Spike et al., 2008). First observed through inadvertent cross-reactivity against a mouse secondary antibody, they were termed “P granules” for their progressive accumulation in the P (posterior) lineage culminating its development in the germline (Figure 1A) (Strome and Wood, 1982). Immediately after fertilization, P granules exist as free-floating and cytoplasmic but later amalgamate around the nucleus, where they extend the nuclear pore complex environment into the cytoplasm and branch into substructures hypothesized to contribute to RNA interference (Strome and Wood, 1982; Hird et al., 1996; Wan et al., 2018; Uebel et al., 2020).

Because the function of P granules has been mysterious, researchers looked to their components for insight. P granules contain proteins associated with RNA binding, degradation, splicing, small RNA-mediated processing, and translational control (Barbee et al., 2002; Shimada et al., 2002; Ogura et al., 2003; Kawasaki et al., 2004; Batista et al., 2008; Gallo et al., 2008; Spike et al., 2008). Additionally, many P granule proteins form multivalent interactions characteristic of condensate formation (Kawasaki et al., 2004; Chen et al., 2020; Aoki et al., 2021). P granules are comprised of at least two distinct phases: an internal liquid-like core characterized by the PGL and GLH proteins and an external, gel-like shell composed of the MEG proteins (Figure 1B). The MEG-phase appears to allow P granules to form a Pickering emulsion, a solid phase-stabilized emulsion, in the cytoplasm (Yang et al., 2017; Putnam et al., 2019; Folkmann et al., 2021).

It was first appreciated that P granules contain specific mRNAs when hybridization experiments demonstrated P granule enrichment of polyA RNA and a paucity of rRNA (Seydoux and Fire, 1994; Pitt et al., 2000; Schisa et al., 2001). Initial efforts identified six mRNAs associated with P granules (Schisa et al., 2001). Of these, a homolog of *Drosophila nanos, nos-2*, emerged as a model transcript illustrating how P granules may function to sequester mRNA for germline-specific translation (Subramaniam and Seydoux, 1999; D'Agostino et al., 2006; Jadhav et al., 2008).

### 2.3 The P Granule Transcriptome is Comprised of Translationally Quiescent Transcripts With Distinct Functional Categories

The model P granule transcript *nos-2 (nanos-2)* accumulates in P granules early in embryogenesis (Subramaniam and Seydoux, 1999) when *nos-2* is translationally repressed by a series of RNA binding protein (RBP) interactions with its 3′UTR (Figure 1A) (D'Agostino et al., 2006; Jadhav et al., 2008). Through its RBP partners, *nos-2* becomes specifically enriched in the posterior (P) lineage as it concentrates within P granules (Subramaniam and Seydoux, 1999; D'Agostino et al., 2006; Jadhav et al., 2008). Once the primordial germ cell has been specified, *nos-2* mRNA emerges from P granules coincident with relief of its translational repression, resulting in NOS-2 protein production exclusively in the germ lineage (Subramaniam and Seydoux, 1999; D'Agostino et al., 2006; Jadhav et al., 2008). For these reasons, the hypothesis emerged that RBPs usher *nos-*2 mRNA to P granules for the purpose of restricting its protein production in both space and time.

**FIGURE 2:**
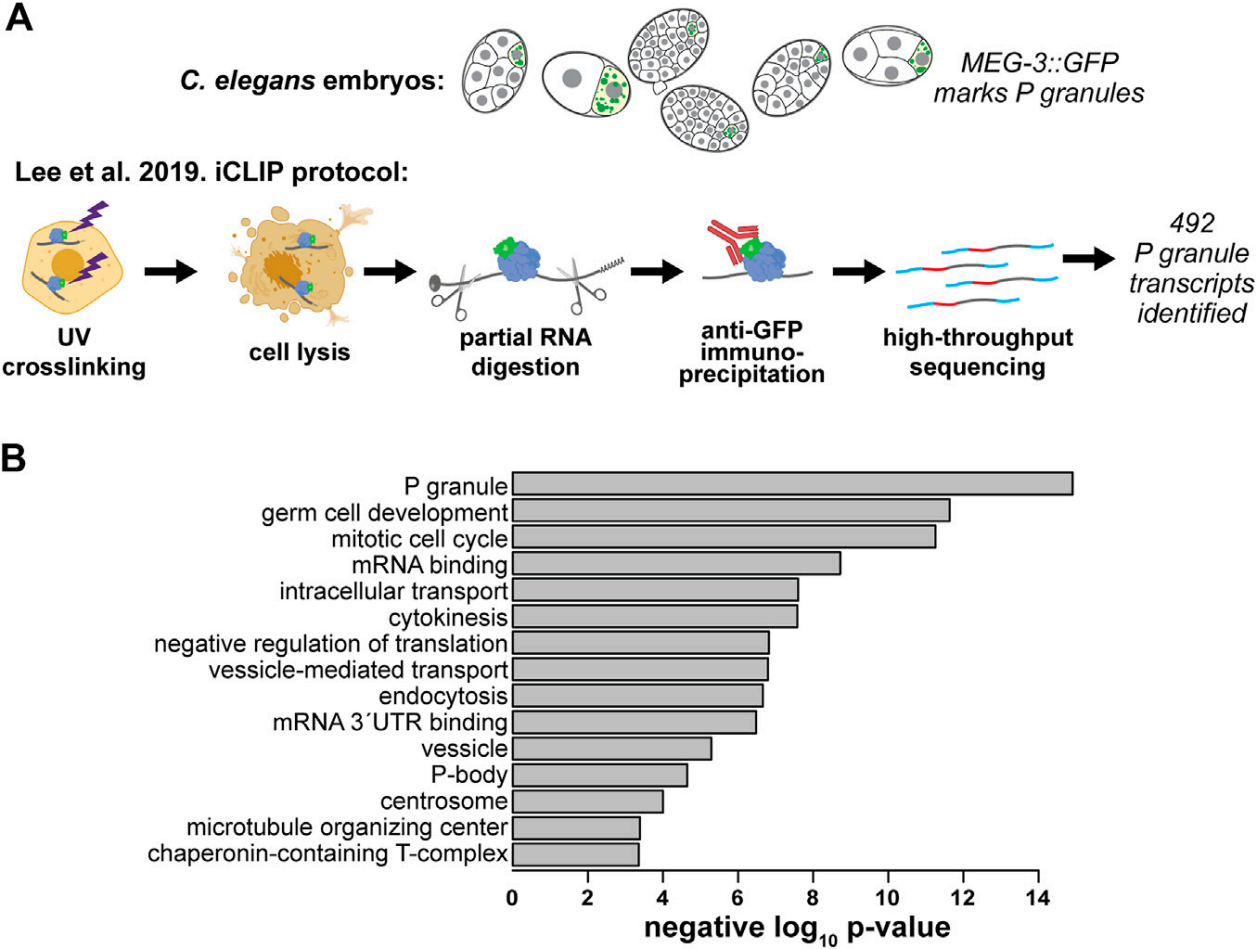
The *C. elegans* P granule transcriptome has been characterized. **(A)** Lee et al. identified 492 transcripts enriched in P granules using an individual nucleotide resolution UV-crosslinking and precipitation (iCLIP) Lee et al. (2020a). **(B)** GO terms enriched in the *C. elegans* P granule transcriptome. We used Lee et al.’s expanded list of 492 MEG-3-associated P granule transcripts to identify enriched categories using the GO: TermFinder (Boyle et al., 2004); Lee et al. (2020a). Transcripts with greater than 10 transcripts per million at any embryonic stage from a previous single-cell resolution RNA-seq study (Hashimshony et al., 2015) were used as a background gene set. The negative log_10_ of each *p* value is shown.

Initially, it was unclear how representative *nos-2* was of P granule transcripts generally (Lee et al., 2020a). However, Lee et al. recently succeeded in characterizing the P granule transcriptome using an iCLIP protocol to target the gel-phase protein, MEG-3::GFP (Figure 2A). This approach was more successful than earlier attempts targeting the liquid phase proteins that failed to enrich RNAs Lee et al. (2020a).

Lee et al.’s expanded atlas of P granule transcripts afforded exploration of their characteristics. The 3′UTRs of messenger RNAs were enriched in the MEG-3 pull down consistent with findings that 3′UTRs are sufficient to direct mRNAs to P granules (Parker et al., 2020). To determine which types of genes associate with MEG-3, we performed gene ontology (GO) on the list of 492 P granule mRNAs identified in Lee et al. (Figure 2B). We found P granule mRNAs are associated with the terms: “P granules,” “germ cell development,” “mRNA binding,” and “negative regulation of translation.” Interestingly, “mitotic cell cycle,” “cytokinesis,” “microtubule organizing center,” and “chaperonin-containing T-complex” terms are also enriched in the P granule transcriptome. Translational repression of these mRNAs may play a role in timing the slow cell cycle of the P lineage leading to their sequestration in P granules (Yokota et al., 2001; Kipreos and van den Heuvel, 2019). P-body-related transcripts were also prevalent, illustrating the similarity between these RNP condensates (see below).

Both Lee et al. and a complementary study by Parker et al. highlighted a key observation—mRNAs that concentrate in P granules are associated with low translational status. Comparing the P granule transcriptome with ribosome profiling data revealed that low ribosome occupancy transcripts enrich in P granules in a sequence-non-specific manner. In contrast, high ribosome occupancy transcripts were depleted from P granules (Lee et al., 2020a).

Whether P granules function to concentrate, asymmetrically localize, surveil, or regulate the translation of their constituent RNAs, they can achieve this function while containing only a minority population of any transcript at any given time. While lowly translated mRNAs are enriched in P granules, only 21–75% of any transcript are within them at any given time (Lee et al., 2020a; Parker et al., 2020). Curiously, many P granule-localized transcripts decay and do not re-emerge for translation representing a complex regulatory control that is not understood. Nonetheless, these findings highlight a perennial question: are mRNA brought to P granules for the purpose of promoting translational repression, or does translational repression promote recruitment to P granules?

### 2.4 Linking Translational Status to P Granules—Repression Leads the Way

The hypothesis that transcripts are brought to P granules to establish translational repression is logical given the paucity of ribosomes in P granules (Schisa et al., 2001). However, observations from stress granules suggest transcripts only maintain association with such condensates if their translational state is already low (Moon et al., 2019).

Single-molecule observations illustrate several circumstances where *nos-2* translation repression occurs independently of P granule accumulation, such as the 1-cell and 2-cell stages of development (Parker et al., 2020). Further, depletion of the RBP PIE-1 (Pharynx and Intestine in Excess) prevents *nos-2* from accumulating in P granules while translation is repressed (Parker et al., 2020). Even when *nos-2* accumulates in P granules, only a fraction of transcripts concentrate there while most remain repressed but dispersed in the cytoplasm (Parker et al., 2020). These findings illustrate that *nos-2* translational repression is independent of P granule accumulation and may occur prior to it. At the transcriptome-wide scale, depletion of MEG-3 and -4 results in P granule dissolution but fails to increase ribosome occupancy of P granule transcripts (Lee et al., 2020a), illustrating that P granule accumulation is not generally required for translation repression. Finally, translation inhibition ectopically promotes recruitment of dispersed transcripts to P granules, implying mRNA accumulation within P granules results from translation regulatory control (Lee et al., 2020a; Parker et al., 2020). Together, these lines of evidence demonstrate that translational repression likely precedes and is sufficient to direct mRNAs to P granules, not the reverse.

It is still possible that P granules reinforce or maintain translational repression after transcripts localize, but that is yet to be determined. This possibility is supported by the observation that ectopic, BoxB-driven recruitment of a translational reporter to P granules causes translational repression (Aoki et al., 2021) when the interaction occurs within adult gonads.

P granules also regulate gene expression through two additional major mechanisms. First, P granules concentrate transcripts in the germ lineage (P lineage) during stages prior to zygotic genome activation when transcription is paused (Seydoux et al., 1996; Tenenhaus et al., 2001). Second, P granules coordinate RNA interference pathways as their disruption leads to aberrations in the pool of endo-siRNAs, progressive loss of RNAi, and transgenerational sterility (Dodson and Kennedy, 2019; Ouyang et al., 2019). Support for the organizational role for P granules comes from similar findings in *Drosophila*. In both *C. elegans* and *Drosophila,* translation repression precedes germ granule accumulation (Gavis and Lehmann, 1994; Rangan et al., 2009; Lee et al., 2020a; Parker et al., 2020), the concentration of components in the germplasm/germ granules is essential for germ cell development (Ephrussi et al., 1991; Kawasaki et al., 1998; Kawasaki et al., 2004; Kistler et al., 2018), and RNA interference components concentrate in germ granules (Huang et al., 2017; Zhang et al., 2018b; Shen et al., 2018). These roles are conserved in germ granules of other organisms, suggesting some germ granule functions are conserved (Voronina et al., 2011).

### 2.5 Germ Granules Serve as Hubs of RNA Regulatory Activity Across Animals

Though the specific names of germ granules, their individual components, and their posited functions are diverse across the animal kingdom, they share several features.

The role of germ granules as hubs of RNA regulatory activity and organization is universal (Chen et al., 2009; Huang et al., 2017; Toombs et al., 2017; Zhang et al., 2018b; Shen et al., 2018). Many of their proteins and RNAs are conserved, with germ granules from all species examined containing Vasa helicases, Argonautes, Xrn1, Nanos protein and RNA, and piRNAs, among others (Voronina et al., 2011). Germ granules also have a clear structural organization. They assemble around nucleating proteins and are typically near mitochondria (Mahowald, 1968; Pitt et al., 2000; Smith et al., 2016; Aoki et al., 2021). Once germ granules nucleate, constituent proteins oligomerize, and RNAs form homotypic clusters, which appear as distinct “domains” within germ granules by microscopy; however, the implications these germ granule domains have on gene regulation are incompletely understood (Trcek et al., 2015; Niepielko et al., 2018; Parker et al., 2020).

Differences do appear to exist. Germ granule nucleating factors diverge quickly at the sequence level and are species-specific (Kulkarni and Extavour, 2017). Moreover, while Argonaute proteins are important for germ granule function, their reported roles differ. In *C. elegans*, the Argonaute PRG-1 is implicated in piRNA regulation and germ granule structure, whereas the *Drosophila* homolog, Aubergine, has an additional role in recruiting mRNA to germ granules through a piRNA-dependent templating mechanism (Vourekas et al., 2016; Suen et al., 2020; Quarato et al., 2021). Notably, in *Drosophila*, some germ-granule-associated mRNAs are translationally repressed outside the granules and only become translated in association with the germplasm or germ granules (Gavis and Lehmann, 1994; Rangan et al., 2009).

Further experimentation may reconcile some apparent differences across species. While germ granule nucleator sequences diverge rapidly over evolutionary time, their functions are conserved. In fact, germ granule nucleators from divergent species are functionally equivalent. When the *Xenopus* germ granule nucleator, Bucky Ball, is replaced with *Drosophila* Oskar, germ granules assemble, and germ cell specification occurs normally (Krishnakumar et al., 2018). This functional equivalence indicates that germ granule nucleators are interchangeable though their primary sequence is not conserved.

Recent studies have demonstrated that germ granules in *C. elegans* are composed of spatially separated condensates with distinct functional roles, such as Z granules, mutator foci, and SIMR foci, among others (Wan et al., 2018; Ouyang et al., 2019; Manage et al., 2020; Uebel et al., 2020). RNA transit from P granules through these different P granule-associated condensates is correlated with distinct outcomes, including inheritance of small RNAs or regulation of exogenous RNAi (Wan et al., 2018; Ouyang et al., 2019; Manage et al., 2020; Uebel et al., 2020). Genetically perturbing the functional organization of P granule-associated condensates causes generational loss of P granules and corresponding sterility (Suen et al., 2020). Similarly, when *Drosophila* Aubergine is lost, germ granules fail to form, resulting in sterility (Sahin et al., 2016). Notably, Aubergine forms a peripheral shell surrounding Tudor labeled germ granules analogous to the various condensates coating P granules (Vo et al., 2019). Thus, the generational loss of P granules when PRG-1 association is lost may be due to a loss of piRNA templated recruitment of mRNA to P granules.

Additionally, while *C. elegans* germ granules are associated with translational repression, some transcripts are known to translate only after a period of association with P granules is relieved. For instance, translational repression of *nos-2* and *Y51F10.2* occurs prior to and then during their association with germ granules (Lee et al., 2020a; Parker et al., 2020). They only undergo translation in later stages around the time when their association with P granules and components of the germ plasm ends. This is similar to the germplasm-associated activation of transcripts in *Drosophila* ^89,90^. Further experiments will determine the degree of conservation among germ granule regulation and organization and which functions are truly distinct to specific animals.

### 2.6 Germ Granules Share Features With Stress Granules and P-Bodies

Beyond the shared roles germ granules play between animals, they also demonstrate similarity to two other cytoplasmic biomolecular condensates, stress granules, and P-bodies.

Stress granules, P-bodies, and germ granules show similarities in their behaviors and compositions while maintaining unique functions (Figure 1B). These organelles are each condensates rich in RNA. Within these condensates, the concentration and conformation of RNAs modulates their formation and dissolution (Teixeira et al., 2005; Schwartz et al., 2013; Lin et al., 2015; Trcek et al., 2015; Saha et al., 2016). They also share protein components. For example, each condensate contains DEAD-box helicases, translation initiation factors, and Argonaute proteins (Gallo et al., 2008; Voronina et al., 2011; Jain et al., 2016; Hubstenberger et al., 2017) while also housing unique proteins that differentiate their functions, such as the PGL and MEG proteins (P granules), GW182 scaffolding protein (P-bodies), or small ribosomal subunits (stress granules) (Liu et al., 2005; Gallo et al., 2008; Buchan and Parker, 2009; Voronina et al., 2011; Seydoux, 2018; Youn et al., 2019; Riggs et al., 2020).

No specific mRNA attribute is known to result in stress granule, P-body, or P granule compartmentalization, excluding a slight bias for longer RNAs (Hubstenberger et al., 2017; Khong et al., 2017; Lee et al., 2020a). These condensates seem only to share the property that they are composed of RNAs that must be post-transcriptionally regulated under various conditions (Chan and Slack, 2006; Batista et al., 2008; Wang and Reinke, 2008; Buchan and Parker, 2009; Protter and Parker, 2016). The primary unifying trait of these condensates is their association with predominantly lowly translated transcripts for either temporary storage or eventual decay (Aizer et al., 2014; Pitchiaya et al., 2019; Parker et al., 2020).

Some transcripts may transfer between germ granules, stress granules, and P-bodies, further demonstrating their shared or coordinated functions in gene regulation (Buchan and Parker, 2009; Protter and Parker, 2016; Hondele et al., 2019). Experiments using purified proteins have demonstrated the directional transfer of transcripts from Dhh1 condensates to Ded1 condensates, the prototypical helicases of yeast P-bodies and stress granules, respectively (Hondele et al., 2019). Similarly, stress granule and germ granule components intermix in *C. elegans* normally and grow concurrently under stress (Gallo et al., 2008; Huelgas-Morales et al., 2016). Meanwhile, P-bodies are known to physically associate and partially overlap with both germ granules and stress granules (Kedersha et al., 2005; Gallo et al., 2008).

Given that stress granules, P-bodies, and germ granules all share components, are associated with low translational output, and can interact and trade constituents, it is surprising that there is no clear model for their coordinated action within cells. Several fascinating questions stand in the way of a clear understanding of their interplay. What is the evolutionary history of these condensates? The existence of stress granules in chloroplast suggests that they may have arisen in prokaryotes (Uniacke and Zerges, 2008); however, it is unclear whether stress granules or P-bodies arose first. Further, while germ granules have significant overlap with stress granules, it is not known whether they were co-opted from stress granules as germ cells developed or if they evolved independently. How does each condensate affect the cellular environment? Considering each condensate contains signaling molecules capable of modulating the intracellular environment, it will be important to explore how each condensate component can impact the condensation, dissolution, composition, and interactions of itself and one another. What factors dictate which RNAs are associated with each condensate? A better understanding of the RBP interactions, post-transcriptional modifications, structures, and conditions distinguishing the RNAs present in each condensate will reveal whether shared RNAs are transferred in an organized, directional manner—suggesting tight regulation of RNA states within each condensate—or whether they are transferred in a stochastic manner—suggesting that their accumulation within condensates is predominantly a consequence of RNA states created through other cellular processes.

## 3 Biomolecular Condensates Mediate Diverse Post-Transcriptional Functions Associated With Active Translation

### 3.1 mRNA can Undergo Translation Within Biomolecular Condensates

Excluding a few exceptions, the emerging theme posits that mRNA within stress granules, P-bodies, and germ granules are of low translation status suggesting that the condensate environment may be refractory to active translation. However, recent discoveries are revealing that translation within these structures occurs and sometimes plays a regulatory role in gene expression.

Indeed, even within stress granules themselves, we now know that some transcripts can be translated. Using the SunTag, an *in vivo* single-molecule translation reporter, Mateju et al. explored the effects of stress response on translation in G3BP1-labeled stress granules. Counter to previous observations demonstrating that transcripts are typically translationally inactive within stress granules^84^, they discovered that the stress response gene, ATF4, is readily translated within stress granules (Mateju et al. 2020). Although it remains true that many RNAs in stress granules fit the canonical model of stress granule association after translation repression, this discovery illustrates that condensates are permissive to diverse forms of post-transcriptional regulation.

**FIGURE 3:**
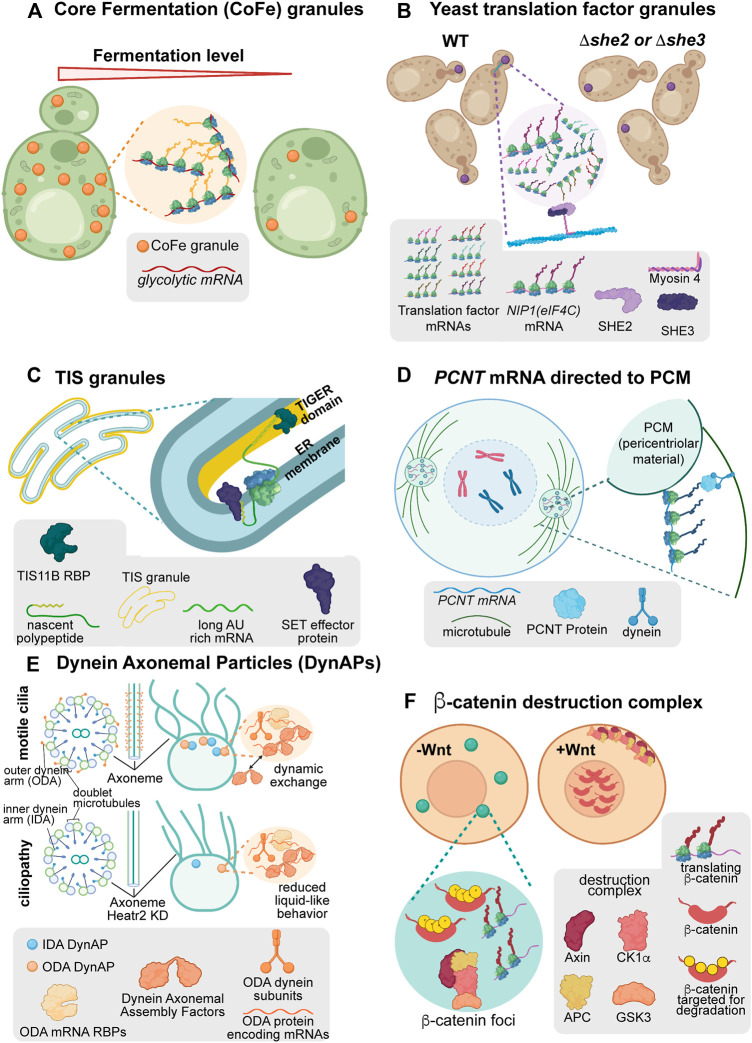
Condensates are associated with diverse forms of post-transcriptional regulation. **(A)** *Saccharomyces cerevisiae* translation factor granules are polarized translation factory condensates associated with translation at regions of growth. When the *SHE2* or *SHE3* genes are removed from cells, these condensates no longer polarize in lab or wild isolate strains. **(B)** Core Fermentation (CoFe) granules in *S. cerevisiae* are translation factory condensates associated with the translation of glycolytic components under fermentative conditions. **(C)** TIS granules in human cell lines are mesh-like condensates interweaved with the ER. In these condensates, TIS11B associates with AU rich elements present in the long 3′UTR isoform of *CD47* to facilitate interaction with its effector protein, SET, upon translation. This condensate-associated interaction promotes increased association of CD47 protein with the cell membrane. **(D)** In human cells, the *PCNT* RNA is actively transported towards the centromere while translating to facilitate rapid incorporation into transient pericentriolar condensates. These condensates support the organization of the pericentriolar material and microtubules to allow mitosis to occur normally. **(E)** Dynein axonemal particles (DynAPs) spatially organize dynein proteins within a condensate environment to promote their appropriate assembly in a “reaction crucible” mechanism. When the condensate environment is disrupted through depletion of Heatr2, Dynein complex assembly is disrupted, axonemal Dynein organization is perturbed, and cilial beating is decreased. **(F)** The ß-catenin destruction complex is a condensate which forms throughout the cell cycle to degrade the constitutively translated ß-catenin protein. Upon induction of Wnt signaling, the components of the destruction complex are modified causing the complex to disassemble and become sequestered at the plasma membrane. When the destruction complex is sequestered, nascent ß-catenin protein can translocate to the nucleus to perform its functions in Wnt signaling.

Among the novel biomolecular condensates associated with active translation are translation factories, locales within the cell where translating ribosomes congregate (Figures 3A,B) (Pizzinga et al., 2019; Morales-Polanco et al., 2021). Some novel condensates serve more nuanced functions, beyond the coordinated, local translational responses mediated by translation factories. These condensates can promote RNA/protein effector interactions (TIS granules, Figure 3C) (Ma and Mayr, 2018), spatiotemporal regulation of organelle assembly (pericentriolar material and axonemal dynein foci/dynein axonemal particles, Figures 3D,E) (Huizar et al., 2018; Sepulveda et al., 2018; Lee et al., 2020b; Fingerhut and Yamashita, 2020; Aprea et al., 2021), and even cell-cycle-dependent protein turnover (ß-catenin destruction complexes, Figure 3F) (Chouaib et al., 2020; Nong et al., 2021a). Here, each of these condensates is discussed as a series of short vignettes to highlight the growing breadth of biophysical behaviors and functional roles for biomolecular condensates.

### 3.2 Translation-Associated “Translation Factory” Condensates

Beyond the occasional translation of mRNA in stress granules, distinct translation-associated mRNA condensates have been reported (Figure 3). Observations have revealed the existence of ribosome-rich, actively translating puncta, or “translation factories,” (Dufourt et al., 2021) such as Glycolytic-bodies (G-bodies) or Core Fermentation (CoFe) granules and translation factor granules, among others (Huizar et al., 2018; Pizzinga et al., 2019; Lee et al., 2020b; Chouaib et al., 2020; Fingerhut and Yamashita, 2020; Fuller et al., 2020; Nong et al., 2021a; Aprea et al., 2021; Morales-Polanco et al., 2021).

Early evidence suggests that many translation factories behave as biomolecular condensates to promote specific regulatory outcomes. In G-bodies, RNAs and intrinsically disordered regions of glycolytic enzymes are required to form gel-like condensates (Jin et al., 2017; Fuller et al., 2020). These RNPs display the hallmark behaviors of condensates: exchange with the surrounding cytoplasm and fusion of separate droplets (Fuller et al., 2020). In CoFe granules, the TRICK assay demonstrated that RNAs are translated in the granules while translation inhibition assays demonstrated active translation is required for glycolytic mRNAs to localize to CoFe granules (Halstead et al., 2016; Morales-Polanco et al., 2021) (Figure 3A). Ultimately, the organization of glycolytic enzymes in translation factory condensates increases the competitive fitness of yeast under hypoxic conditions compared to cells without the condensate organization (Fuller et al., 2020). Notably, CoFe granules have also been observed in HeLa cells, suggesting this may be a conserved mechanism for regulating glycolysis pathways (Morales-Polanco et al., 2021).

Similarly, translation factor RNAs translate in translation factories in yeast (Pizzinga et al., 2019) (Figure 3B). These translation factories, termed translation factor granules, are a heterogeneous composition of initiation, elongation, and termination factor RNAs, which polarize towards the budding daughter cell under normal growth conditions. Supporting condensate behavior, these translation factories disperse upon treatment with aliphatic alcohol and fuse with P-bodies upon glucose deprivation. As with G-bodies/CoFe granules, translation factor granules contain actively translating RNAs as confirmed by TRICK (Halstead et al., 2016). When the polarization of translation factor granules is disrupted, as with impairment of the transport factors She2p or She3p, yeast strains fail to undergo filamentous growth. This demonstrates a role for these translation factories in regulating cell polarization and competitive fitness in the wild.

The organization of RNAs into translation factory condensates appears thematic in its capacity to promote competitive fitness and promote cellular viability. Translation-associated condensates are also being implicated in the misregulation of translation. Recent data has provocatively suggested that solid-like amyloid condensates may facilitate stress-response-induced translation in the nucleus to preserve cell viability (Theodoridis et al., 2021). While more comprehensive screening is needed to define the breadth and functions of translation factory-associated RNAs, their regulation is interesting nonetheless. Understanding what coordinates the spatial organization of mRNAs and their relationship to the translation state within these condensates will provide a deeper understanding of the mechanisms regulating gene expression.

### 3.3 TIS Granule Condensates Mediate Protein-Effector Interactions

The ER is associated with the local translation of secreted or membrane proteins (Walter and Blobel, 1981; Walter and Johnson, 1994; Ast et al., 2013). Recent discoveries have added complexity to this model, however. A novel ER-associated condensate called the TIS granule facilitates local translation and protein-effector assembly adjacent to the ER (Ma and Mayr, 2018; Ma et al., 2021).

TIS granules appear as an extraluminal, space-filling condensate interleaved with the ER containing the RBP TIS11B and its RNA targets. TIS granules form a mesh-like rather than spherical structure and can only undergo partial fusion events suggesting they behave as a gel-like condensate (Ma et al., 2021). Even so, these structures behave with liquid-like dynamics, demonstrating rapid recovery of TIS11B fluorescence after photobleaching. This paradoxical behavior is mediated through extensive RNA-RNA interactions. By forming RNA interaction networks, TIS granules retain RNA while allowing interacting proteins such as TIS11B to dynamically flux through the condensate environment (Ma et al., 2021).

Within TIS granules, TIS11B binds the transcript CD47 at AU-rich elements present only in the longer of two alternatively-spliced 3′UTR isoforms resulting in its localization to the TIGER (TIs Granule ER) domain. This distinct environment promotes CD47 protein interaction with the effector protein, SET, in a splice-variant-specific manner. The extended mesh-like structure of TIS granules may provide an expanded surface area for these interactions to occur (Ma and Mayr, 2018). Ultimately, this condensate-mediated complex formation promotes increased membrane localization of the CD47 protein compared to protein translated from the short, non-TIS granule localized CD47 isoform. The TIS granule demonstrates how transcript localization in condensates can mediate specific protein-protein interactions and fate directed by untranslated mRNA elements.

### 3.4 Local Translation of *PCNT* RNA Correlates Rapid Condensate-Like Growth of the Pericentriolar Material

Post-transcriptional regulation in condensates can transcend the protein-effector interactions observed in TIS granules and support the organization of entire organelles. One example is the cotranslational localization of *PCNT* mRNA to the pericentriolar material (PCM).

At the onset of mitosis, the PCNT protein promotes condensation of PCM surrounding the centrosome (Woodruff et al., 2017; Sepulveda et al., 2018). This condensation is in part organized by localization of *PCNT* mRNA to centrosomes in a translation- and dynein-dependent manner during centrosome maturation (Sepulveda et al., 2018). *PCNT* mRNA localization combats the kinetic challenge of transporting and synthesizing this large protein (3336 amino acids in humans) to direct PCM and centrosome formation during the short period of early mitosis.

Upon localization, PCNT protein forms dynamic pericentriolar granules. Supporting their condensate behavior, these granules are sensitive to aliphatic alcohols *in vivo.* Additionally, the N-terminal low complexity region of the protein displays rapid recovery after photobleaching, has a distinct phase boundary, and granules can coalesce and deform (Jiang et al., 2021). The PCNT condensates can then recruit other PCM components and ultimately promote microtubule nucleation. Over a short time, the PCM appears to “mature” into more solid-like structures, posited to withstand the extensive forces of mitosis (Woodruff et al., 2015; Woodruff et al., 2017). This dynamic but transient condensate behavior of PCNT granules in organizing the PCM illustrates that biomolecular condensates can support the formation of entire organelles over brief periods.

### 3.5 Assembly of Axonemal Dynein Within Condensates is Required for Cilial Beating

Dynein complexes comprising the primary minus-end-directed microtubule motor are essential for the normal movement of cilia. Axonemal dynein foci, otherwise known as Dynein Axonemal Particles (DynAPs), are essential for assembling these dynein complexes (Huizar et al., 2018; Lee et al., 2020b; Fingerhut and Yamashita, 2020; Aprea et al., 2021). DynAPs spatially organize inner and outer dynein arm components and their partners to promote proper assembly and subsequent axonemal distribution of dynein complexes in *Xenopus* multiciliated cells.

These granules demonstrate standard condensate behaviors. DynAPs components recover rapidly from photobleaching, readily undergo fission and fusion, and even share some, but not all, components with stress granules (Huizar et al., 2018). The condensate behavior of DynAPs supports the assembly of axonemal dynein complexes through a “reaction crucible” mechanism. Dynein components are maintained stably within the DynAPs, while dynein axonemal assembly factors and other chaperones can flux through the condensate to promote functional complex organization (Huizar et al., 2018). When the condensates are made less mobile by depletion of the DynAP protein Heatr2, ciliary beating decreases, suggesting that the dynamic exchange of assembly factors in DynAPs is essential for proper dynein complex assembly (Huizar et al., 2018). In turn, loss of axonemal dynein causes defects in ciliary beating, cell motility, and in some instances, sterility (Fingerhut and Yamashita, 2020; Aprea et al., 2021). These defects demonstrate the importance of condensates in promoting proper complex assembly, preventing inappropriate interactions between complexes, and the ultimate function of organelles and the cell.

### 3.6 The ß-Catenin Destruction Complex

One particularly striking form of condensate-mediated post-transcriptional regulation is the ß-catenin “destruction complex.” The destruction complex is a condensate formed through the multivalent interaction of Axin and APC proteins (Nong et al., 2021a). *In vivo* Axin has been shown to have condensate behavior as evidenced by its fusion and recovery after photobleaching, while further support comes from its salt sensitivity and concentration-dependent phase-separation (Nong et al., 2021b).

The striking aspect of the destruction complex is that ß-catenin mRNA perpetually translates throughout the cell cycle within the destruction complex, but the ß-catenin protein is rapidly degraded in these condensates. Within the destruction complex, ß-catenin phosphorylation by Axin-recruited kinases promotes subsequent proteasomal degradation. However, upon the activation of Wnt signaling, destruction complex proteins are recruited to the cell membrane (Chouaib et al., 2020). The sequestration of destruction complex components dissolves the condensate (Chouaib et al., 2020). At this point, ß-catenin safely transits to the nucleus to perform its signaling functions. This constant cycle of protein synthesis and degradation allows for the rapid and specific response required for functional Wnt signaling as the cell cycle progresses while preventing the dominant-negative effects that occur in the presence of constitutive ß-catenin protein (Nong et al., 2021a). This complex regulation demonstrates how concentrating transcripts within condensates can overcome kinetic challenges to meet the needs of the cell.

Overall, cytoplasmic biomolecular condensates represent dynamic environments. Germ granules, stress granules, and P bodies are enriched for translationally inactive transcripts, but newly discovered roles for condensates in post-transcriptional regulation adds complexity to these models with many examples of condensates that form in association with active translation or for the purpose of regulating translational outcomes. Even condensates long thought to house translationally repressed RNAs, such as stress granules, can host the translation of a subset of their constituents. As a result, the functional purpose of organizing RNA and protein into biomolecular condensates is an active field of research with many remaining questions.

## 4 Conclusion

Though the exact functions of many biomolecular condensates remain elusive, the fact remains: mRNA localization, transcript function, and translational control are intimately linked to these phase-separated organelles. It is advantageous to translate proteins when and where they are required and to maintain translational repression when translation would be hazardous to cell viability. It is increasingly apparent that mRNA accumulation within biomolecular condensates is integral to this regulation.

Many unanswered questions remain. A key challenge will be to determine where RNA condensation itself is essential. In some classic examples, such as the germ granule RNAs in *Drosophila*, localization is clearly important in development as loss of germ granules causes severe morphogenic phenotypes and lethality (Voronina et al., 2011). However, the functional effects of removing any particular transcript from a condensate are challenging to study. Disruption of condensates can result in the misregulation of the entire organelle. Even when experimenting with one transcript, the localization and translation regulatory elements are often difficult or impossible to separate, causing pleiotropic effects when inducing their mislocalization (D'Agostino et al., 2006; Jadhav et al., 2008; Parker et al., 2020; Paquin et al., 2007; Chartrand et al., 2002; Hüttelmaier et al., 2005). Thus, it is uncertain whether RNA condensation is a causative, redundant, reinforcing, or symptomatic effect of regulatory control in many instances.

Another challenge is understanding the relationships between the cytoplasmic RNP condensates such as stress granules, P-bodies, and germ granules given that many of their protein and RNA components overlap (Figure 1B). Do they share an evolutionary origin? Do they communicate with one another? Are there pathways that transfer mRNAs from condensate to condensate? How do these largely repressive condensates relate to translation-associated condensates like translation factories? Increased application of *in vivo* imaging will reveal which mRNAs are true long-term residents of condensates, which are merely migrating through them, and how this correlates with their translational regulation.

Additionally, it will be interesting to continue exploring the structural role of RNA itself in mediating phase separation. Several studies have demonstrated that mRNA can scaffold condensates, such as *RPS28B,* which recruits its own protein to scaffold P-bodies, and the *Ashbya gossypia* mRNAs *CLN3*, *BNI1*, and *SPA2* that assist in determining the specificity of Whi3 droplets (Langdon et al., 2018a; Fernandes and Buchan, 2020). Incredibly, some mRNA sequences appear to have intrinsic localization cues. The *BglG* mRNA in *E. coli* may localize to cell membranes through a PolyU tract, which can interact electrostatically with membranes *in vitro* (Nevo-Dinur et al., 2011; Kamat et al., 2015). Furthermore, some RNAs form homotypic clusters *in vivo* or self-segregate *in vitro* (Little et al., 2015; Trcek et al., 2015; Langdon et al., 2018b; Tauber et al., 2020). Understanding the fundamental physics of RNA interactions with cellular components will inevitably provide insight into how condensates form, their internal dynamics, and their ultimate functional effects.

Though a complete understanding of condensates remains unclear, their defects can result in pronounced phenotypes and human disease. Defects in mouse germ granule components impair spermatogenesis (Lehtiniemi and Kotaja, 2018). In *Drosophila* and *Xenopus,* defects in germ granules prevent germline development, while in *C. elegans*, loss of P granules causes germline transcriptomic changes and can result in immediate, temperature-sensitive, or multi-generational progressive sterility (Kawasaki et al., 1998; Wang and Reinke, 2008; Dodson and Kennedy, 2019; Ouyang et al., 2019). Stress granules regulate nucleocytoplasmic transport, which misfunctions in amyotrophic lateral sclerosis (ALS) and frontotemporal dementia (FTD) (Fan and Leung, 2016; Zhang et al., 2018a). These transport defects are rescued by perturbing stress granule components (Zhang et al., 2018a). Neurological disorders are also associated with P-body dysregulation as mutations in the DDX6 helicase prevent proper assembly of P-bodies and ultimately result in intellectual disability in humans (Balak et al., 2019). Further, mutants for the P-body proteins DCP-1 and DCP-2 exhibit phenotypes in pattern-triggered immunity resulting in pathogen susceptibility (Yu et al., 2019).

By understanding the components and mechanisms cells use to organize RNA and regulate local translation we can begin to better design experiments. In the lab, developing nuanced genetic tools to control the temporal availability of proteins in living cells could provide new tunable or inducible expression systems. Further, identifying cis-acting elements sufficient for sequestration of transcripts away from their usual destination will allow for dissecting the functions of RNA localization *in vivo.* An improved understanding will also lead to advances in the treatments of human diseases. In medicine, understanding the mechanisms underlying misregulation of condensates implicated in neurological disorders can impact human health by supporting the search for treatments. Perhaps more importantly, it may reveal the underlying genetics and environmental conditions that contribute to the progression of these diseases allowing for more preventative measures to be taken.

As this field matures, insights will continue to emerge. The theme that multiple modes of mRNA regulation can occur concurrently within condensates is likely to continue. The interrelatedness between mRNA localization, translation regulation, decay, and small RNA-mediated regulation will continue to come into focus. Discoveries of highly specialized biomolecular condensates are likely to accelerate as we determine how the biophysical properties of these structures impact the biochemistry of mRNA regulatory control. Finally, the linkages between coordinated translational control at each distinct level of translation initiation, elongation, termination, and recycling are all likely to be important. The field is rich for potential discoveries as mRNA condensation and translation regulatory control emerge from a niche field, studied in a few systems, to a generalizable feature of cell biology.
